# ﻿Three new species of the genus *Ischnothyreus* Simon, 1893 and the discovery of the male of *I.linzhiensis* Hu, 2001 from Tibet, China (Araneae, Oonopidae)

**DOI:** 10.3897/zookeys.1152.100341

**Published:** 2023-03-09

**Authors:** Yanfeng Tong, Dongju Bian, Shuqiang Li

**Affiliations:** 1 Life Science College, Shenyang Normal University, Shenyang 110034, Liaoning, China Shenyang Normal University Shenyang China; 2 Key Laboratory of Forest Ecology and Management, Institute of Applied Ecology, Chinese Academy of Sciences, Shenyang 110016, China Institute of Applied Ecology, Chinese Academy of Sciences Shenyang China; 3 Institute of Zoology, Chinese Academy of Sciences, Beijing 100101, China Institute of Zoology, Chinese Academy of Sciences Beijing China

**Keywords:** Distribution, goblin spiders, morphology, taxonomy

## Abstract

Four species of the genus *Ischnothyreus* Simon, 1893 from Tibet, China are recognised, including three new species, *I.caoqii***sp. nov.** (female), *I.metok***sp. nov.** (female), and *I.pome***sp. nov.** (male and female). Males of *Ischnothyreuslinzhiensis* Hu, 2001 are discovered for the first time since its description. Descriptions, diagnoses, and photographs of the four species are provided.

## ﻿Introduction

Oonopidae is a diverse spider family with 1888 extant described species in 115 genera (WSC 2023). They have a nearly worldwide distribution, occurring mainly in the leaf litter, under bark, and in the tree canopy (Jocqué and Dippenaar-Schoeman 2006; Ubick and Dupérré 2017). The genus *Ischnothyreus* Simon, 1893 is one of the most speciose genera of Oonopidae, with 121 extant species mainly distributed in the Old World (WSC 2023).

The oonopid spiders of Tibet have been poorly studied. Hu (2001) reported two new species from Nyingchi, Tibet, i.e., *Gamasomorphalinzhiensis* Hu, 2001, and *Ischnothyreuslinzhiensis* Hu, 2001. Cheng et al. (2021) reported one new genus and two new species from Nyingchi, Tibet, i.e., *Paramolotrapome* Tong & Li, 2021, and *Paramolotrametok* Tong & Li, 2021. In this paper three new species of the genus *Ischnothyreus*, collected from Tibet, are reported and a detailed re-description of *I.linzhiensis* Hu, 2001 is provided including the first male.

## ﻿Materials and methods

The specimens were examined using a Leica M205C stereomicroscope. Details were studied under an Olympus BX51 compound microscope. Vulvae were cleared in lactic acid. Photomicroscope images were made with a Canon EOS 750D zoom digital camera (18 megapixels) mounted on an Olympus BX51 compound microscope. Photos were stacked with Helicon Focus 6.7.1 and processed in Adobe Photoshop CC 2020. For scanning electron microscopy (**SEM**), specimens were air-dried, sputter-coated using IXRF SYSTEMS, and imaged with a Hitachi TM3030 SEM. All measurements were taken using an Olympus BX51 compound microscope and are in millimeters. All specimens are preserved in 75% ethanol. The type material is deposited in Shenyang Normal University (**SYNU**) in Shenyang, China.

The following abbreviations are used in the text and figures: **a** = apodeme; **ALE** = anterior lateral eye; **ca** = conical apophysis; **css** = chestnut-shaped structure; **dm** = dorsal membrane; **flp** = flag-like process; **llp** = leaf-like projection; **nlm** = needle-like membrane; **PLE** = posterior lateral eye; **PME** = posterior median eye; **rlp** = ridge-like protuberance; **sls** = snout-like structure; **ssd** = semicircle-shaped depression; **sss** = semicircle-shaped structure; **stp** = strong, tooth-like projection; **vp** = ventral projection; **vpr** = ventral protuberance; **wd** = winding duct.

## ﻿Taxonomy

### ﻿Family Oonopidae Simon, 1890

**Genus *Ischnothyreus* Simon, 1893**

#### ﻿ Ischnothyreus caoqii sp. nov.

Taxon classificationAnimaliaAraneaeOonopidae

ADD55BE6-37BA-5875-9A6C-8A6745781802

https://zoobank.org/1BFB2DB2-6119-46E4-B55E-BD37638576BC

Fig. 1A–I

##### Type material.

***Holotype*** ♀ (SYNU-508): China, Tibet, Nyingchi, Pome County, road to Metok County, 80 K; 29°39.897'N, 95°29.963'E; 2140±5 m; 10.VIII.2013; Qi Cao leg.

##### Diagnosis.

**Figure 1.**
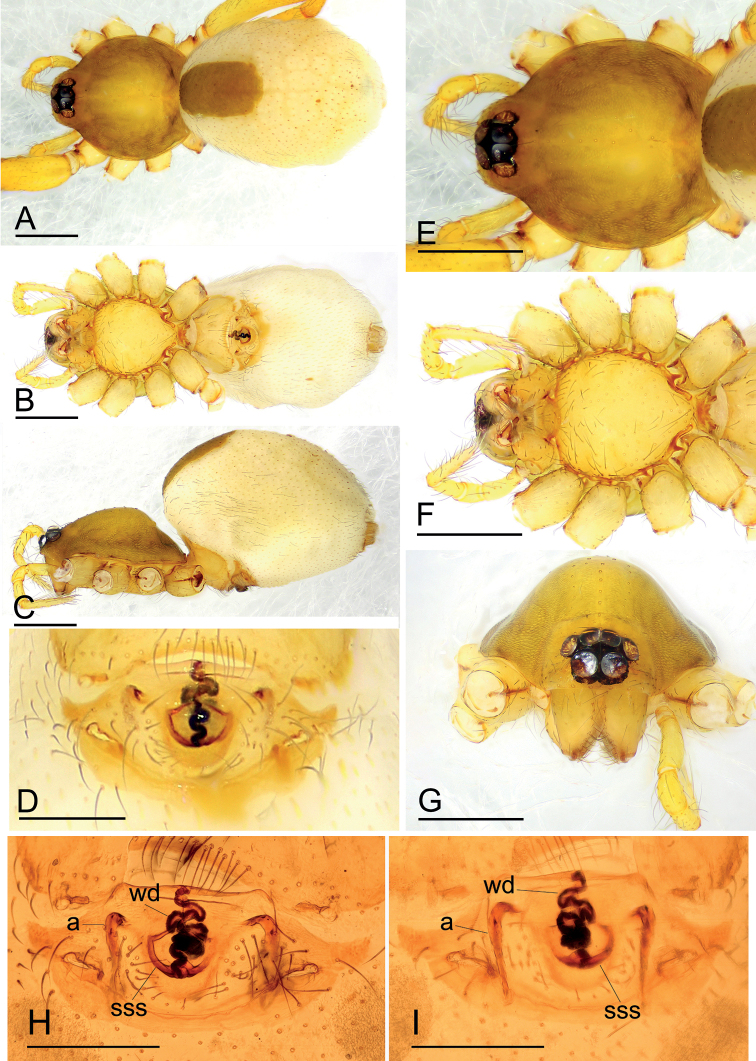
*Ischnothyreuscaoqii* sp. nov., female holotype **A–C** habitus in dorsal, ventral and lateral views **D** epigastric region, ventral view **E–G** prosoma, dorsal, ventral and anterior views **H, I** endogyne, ventral and dorsal views (cleared in lactic acid). Abbreviations: a = apodeme; sss = semicircle-shaped structure; wd = winding duct. Scale bars: 0.4 mm (**A–C, E–G**); 0.2 mm (**D, H, I**).

The new species is similar to *I.jianglangi* Tong & Li, 2020 in the size of the abdominal scuta, but can be distinguished by the large, semicirculared structure of the endogyne and the simple winding duct (Fig. 1H, I) vs. a triangular structure and a complex winding duct (see Tong et al. 2020: fig. 17A, B).

##### Description.

**Female (holotype). *Body***: habitus as in Fig. 1A–C; body length 1.96. ***Carapace***: 0.94 long, 0.82 wide; brownish, with a yellowish center, broadly oval in dorsal view, pars cephalica strongly elevated in lateral view, surface of elevated portion of pars cephalica smooth, sides finely reticulate, lateral margin straight, smooth (Fig. 1E). ***Clypeus***: straight in frontal view, ALE separated from edge of carapace by less than radius (Fig. 1G). ***Eyes***: ALE largest, ALE circular, PME squared, PLE oval; posterior eye row recurved from above; ALE touching, ALE-PLE touching (Fig. 1E, G). ***Sternum***: longer than wide, pale orange (Fig. 1F). ***Mouthparts***: chelicerae, endites, and labium orange; chelicerae and endites unmodified. ***Abdomen***: 1.05 long, 0.73 wide; dorsal scutum well sclerotized, pale orange, covering 1/3 of abdomen width and approximately 1/3 of abdomen length; epigastric scutum well sclerotized, pale orange; postgastric scutum hexagonal. ***Legs***: pale orange, femur I with two prolateral spines, tibia I with four pairs, metatarsus I with two pairs of long ventral spines. Leg II spination similar to leg I, except femur with only one prolateral spine. Legs III and IV spineless. ***Endogyne***: winding duct complex, strongly convoluted, ending in semicircle-shaped structure (Fig. 1H, I).

**Male.** Unknown.

##### Etymology.

The species is named after Mr. Qi Cao, the collector of the type specimens; noun in genitive case.

##### Distribution.

Known only from the type locality.

#### ﻿ Ischnothyreus linzhiensis

Taxon classificationAnimaliaAraneaeOonopidae

Hu, 2001

789AD17F-C440-5F21-A5E5-7D97E4DA39CA

**Figure 2.**
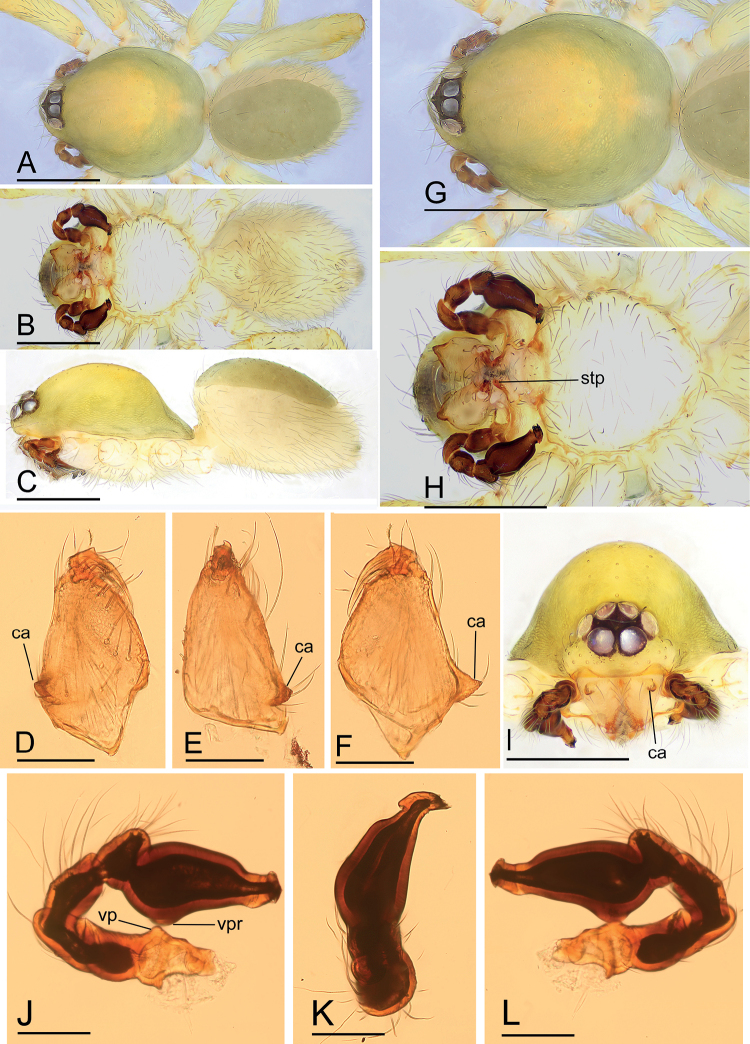
*Ischnothyreuslinzhiensis* Hu, 2001, male **A–C** habitus in dorsal, ventral and lateral views **D–F** left chelicera, anterior, lateral and posterior views **G–I** prosoma, dorsal, ventral and anterior views **J–L** left palp, prolateral, dorsal and retrolateral views. Abbreviations: ca = conical apophysis; stp = strong, tooth-like projection; vp = ventral projection; vpr = ventral protuberance. Scale bars: 0.4 mm (**A–C, G–I**); 0.1 mm (**D–F, J–L**).

**Figure 3.**
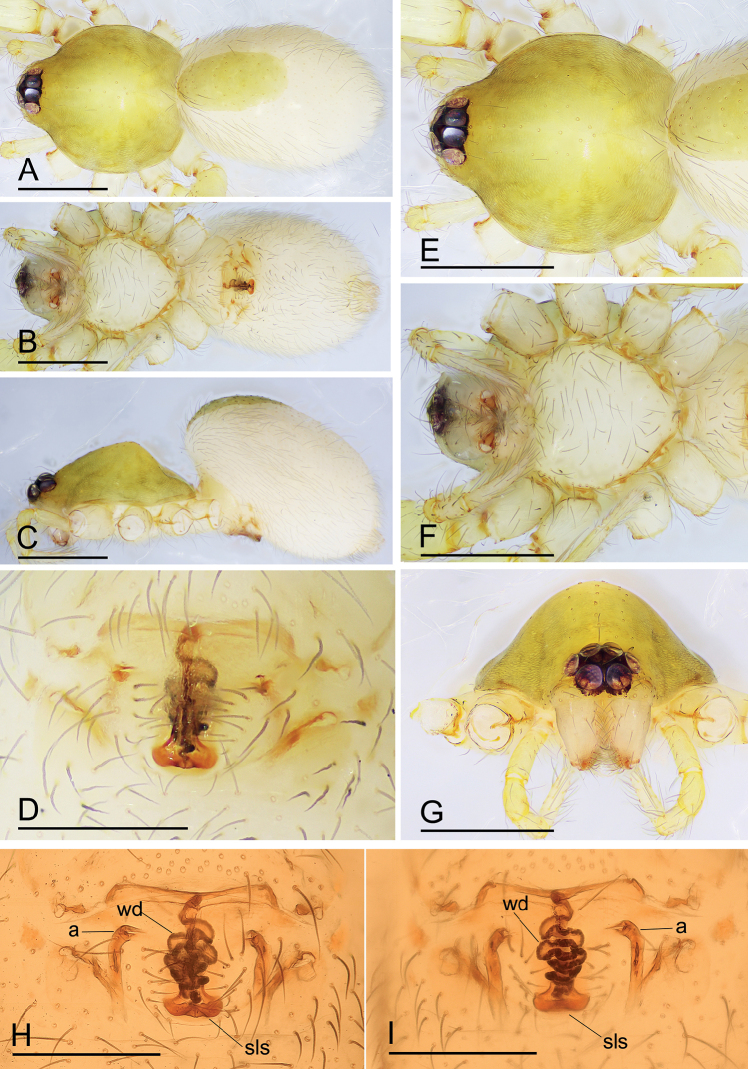
*Ischnothyreuslinzhiensis* Hu, 2001, female **A–C** habitus in dorsal, ventral and lateral views **D** epigastric region, ventral view **E–G** prosoma, dorsal, ventral and anterior views **H, I** endogyne, ventral and dorsal views (cleared in lactic acid). Abbreviations: a = apodeme; sls = snout-like structure; wd = winding duct. Scale bars: 0.4 mm (**A–C, E–G**); 0.2 mm (**D, H, I**).

**Figure 4.**
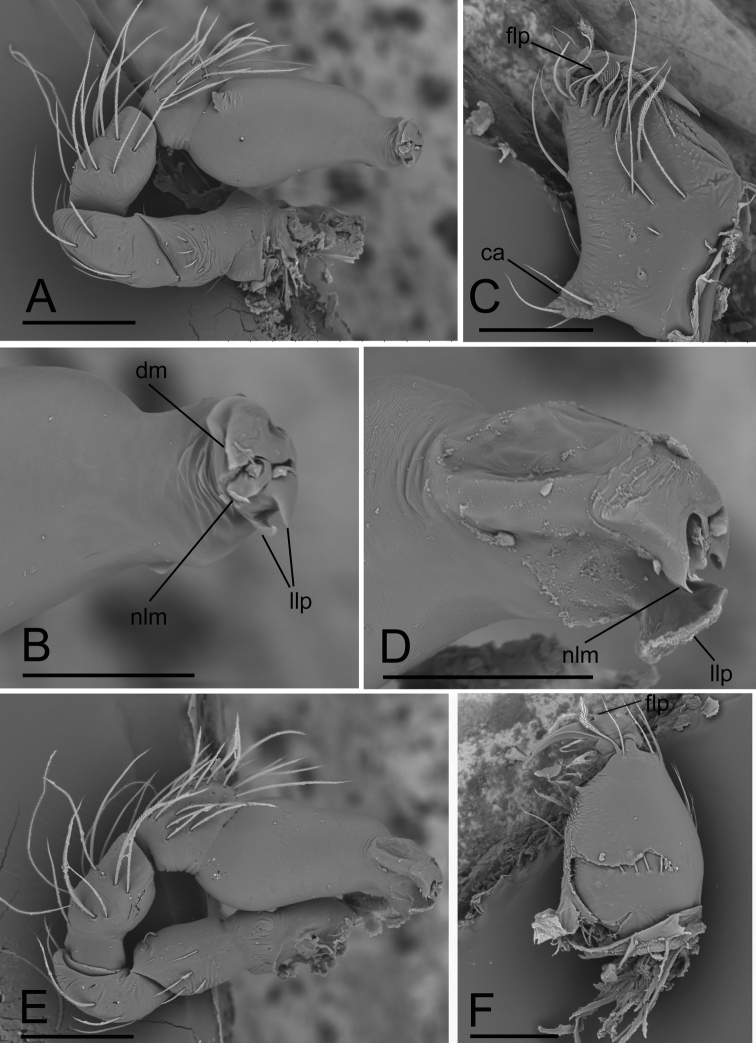
*Ischnothyreuslinzhiensis* Hu, 2001, male **A–C**SEM; *Ischnothyreuspome* sp. nov., male holotype **D–F**SEM**A, E** left palp, prolateral view **B, D** distal end of bulb, prolateral view **C, F** left chelicerae, anterior and posterior views. Abbreviations: ca = conical apophysis; dm = dorsal membrane; flp = flag-like process; llp = leaf-like projection; nlm = needle-like membrane. Scale bars: 0.1 mm (**A, C, E, F**); 0.05 mm (**B, D**).

Figs 2A–L , 3A–I , 4A–C

##### Material examined.

7♂, 9♀ (SYNU-509-524): China, Tibet, Nyingchi, Pome County, Yigong Town, Kaduo Village, 30°07.515'N, 95°01.928'E; 2072±3 m; 10.VIII.2013; Qi Cao leg.

##### Diagnosis.

This species is similar to *I.serpentinum* Saaristo, 2001 in the morphology of the winding duct and the size of the abdominal scuta, but can be distinguished by the large, snout-like structure of the endogyne (Fig. 3H, I) vs. a small posterior process (see Richard et al. 2016: fig. 75E), and the conical apophysis of the male cheliceral anterior face and the flag-like sclerotized process of the fang base (Figs 2D–F, 4C) vs. unmodified chelicerae (see Richard et al. 2016: fig. 70E).

##### Description.

**Male (SYNU-509). *Body***: habitus as in Fig. 2A–C; body length 1.64. ***Carapace***: 0.88 long, 0.65 wide; yellow, oval in dorsal view, pars cephalica strongly elevated in lateral view, surface of elevated portion of pars cephalica smooth, sides finely reticulate, lateral margin straight, smooth (Fig. 2G). ***Clypeus***: straight in frontal view, ALE separated from edge of carapace by their diameter (Fig. 2I). ***Eyes***: ALE largest, ALE circular, PME squared, PLE oval; posterior eye row recurved from above; ALE touching, ALE-PLE touching (Fig. 2G, I). ***Sternum***: as long as wide, pale orange (Fig. 2H). ***Mouthparts***: chelicerae, endites and labium yellow; chelicerae straight, anterior face with conical apophysis, base of fangs with large flag-like sclerotized process, fang groove with a few small and one larger denticles (Figs 2D–F, 3C); anteromedian tip of endites with one strong, tooth-like projection (Fig. 2H). ***Abdomen***: 0.76 long, 0.44 wide; dorsal scutum dark brown, covering 4/5 of abdomen width and approximately 5/6 of abdomen length, not fused to epigastric scutum; postgastric scutum covering ~ 2/3 of abdomen length. ***Legs***: pale orange, femur I with two prolateral spines, tibia I with four pairs, metatarsus I with two pairs of long ventral spines. Leg II spination similar to leg I, except femur with only one prolateral spine. Legs III and IV spineless. ***Palp***: trochanter with ventral projection; bulb with one ventral protuberance, distal end of bulb stout, with dorsal membrane, needle-like membrane and two leaf-like projections (Figs 2J–L, 4A, B).

**Female (SYNU-516).** Same as male except as noted. ***Body***: habitus as in Fig. 3A–C; body length 1.76. ***Carapace***: 0.72 long, 0.67 wide. ***Mouthparts***: chelicerae and endites unmodified. ***Abdomen***: 1.05 long, 0.67 wide; dorsal scutum very small; epigastric scutum well sclerotized, orange; postgastric scutum widely hexagonal, only around epigastric furrow. ***Endogyne***: winding duct complex, strongly convoluted, ending in large, snout-like structure (Fig. 3H, I).

##### Distribution.

China (Tibet: Nyingchi).

#### ﻿ Ischnothyreus metok sp. nov.

Taxon classificationAnimaliaAraneaeOonopidae

BBEDC6A0-129D-5877-B3BB-B8144D75F0D8

https://zoobank.org/455D6D15-EE9C-480D-A82B-84813FF407C2

Fig. 5A–I

##### Type material.

***Holotype*** ♀ (SYNU-525): China, Tibet, Nyingchi, Metok County, Metok Town, Countryside Tour, 29°19.087'N, 95°18.876'E, 1280±5 m, 4.VIII.2013, Qi Cao leg.

##### Diagnosis.

**Figure 5.**
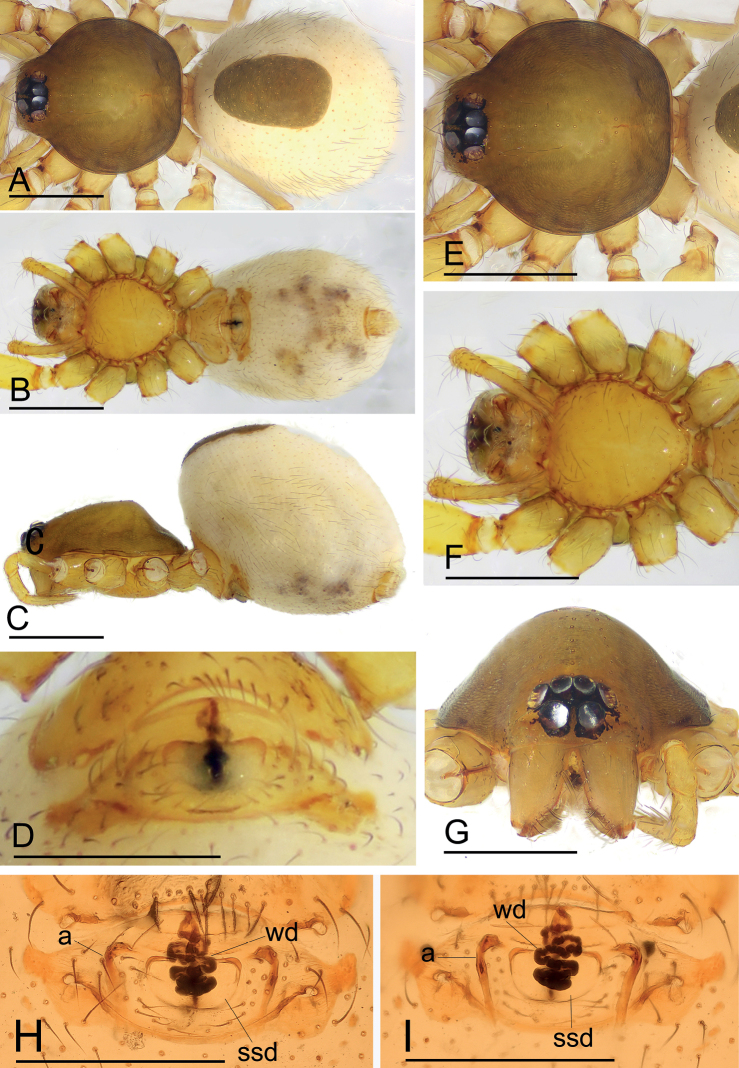
*Ischnothyreusmetok* sp. nov., female holotype **A–C** habitus in dorsal, ventral and lateral views **D** epigastric region, ventral view **E–G** prosoma, dorsal, ventral and anterior views **H, I** endogyne, ventral and dorsal views (cleared in lactic acid). Abbreviations: a = apodeme; ssd = semicircle-shaped depression; wd = winding duct. Scale bars: 0.4 mm (**A–C, E–G**); 0.2 mm (**D, H, I**).

The new species is similar to *I.comicus* Edward & Harvey, 2014 in the size of the abdominal scuta, but can be distinguished by the semicircle-shaped depression of the endogyne (Fig. 5H) vs. a smile-shaped depression (see Edward and Harvey 2014: figs 13I, 14F).

##### Description.

**Female (holotype). *Body***: habitus as in Fig. 5A–C; body length 1.64. ***Carapace***: 0.77 long, 0.67 wide; dark brown, broadly oval in dorsal view, pars cephalica strongly elevated in lateral view, surface of elevated portion of pars cephalica finely reticulate, sides strongly reticulate, lateral margin straight, smooth (Fig. 5E). ***Clypeus***: straight in frontal view, ALE separated from edge of carapace by less than their radius (Fig. 5G). ***Eyes***: ALE largest, ALE circular, PME squared, PLE oval; posterior eye row procurved from above; ALE separated by less than their radius, ALE-PLE touching (Fig. 5E, G). ***Sternum***: longer than wide, pale orange (Fig. 5F). ***Mouthparts***: chelicerae, endites and labium yellow; chelicerae and endites unmodified**. *Abdomen***: 0.88 long, 0.65 wide; dorsal scutum well sclerotized, dark brown, covering 1/3 of abdomen width and approximately 1/3 of abdomen length, not fused to epigastric scutum; epigastric scutum well sclerotized, yellow; postgastric scutum widely hexagonal. ***Legs***: femur I with two prolateral spines, tibia I with four pairs, metatarsus I with two pairs of long ventral spines. Leg II spination similar to leg I, except femur with only one prolateral spine. Legs III and IV spineless. ***Endogyne***: with large, semicircle-shaped depression; winding duct complex, strongly convoluted (Fig. 5H, I).

**Male.** Unknown.

##### Etymology.

The specific name is a noun in apposition taken from the type locality.

##### Distribution.

Known only from the type locality.

#### ﻿ Ischnothyreus pome sp. nov.

Taxon classificationAnimaliaAraneaeOonopidae

6489A94F-F7B5-5B9B-9BA6-3309BA106BFB

https://zoobank.org/89199E5A-66CB-4CA0-8396-6000B96A81EA

**Figure 6.**
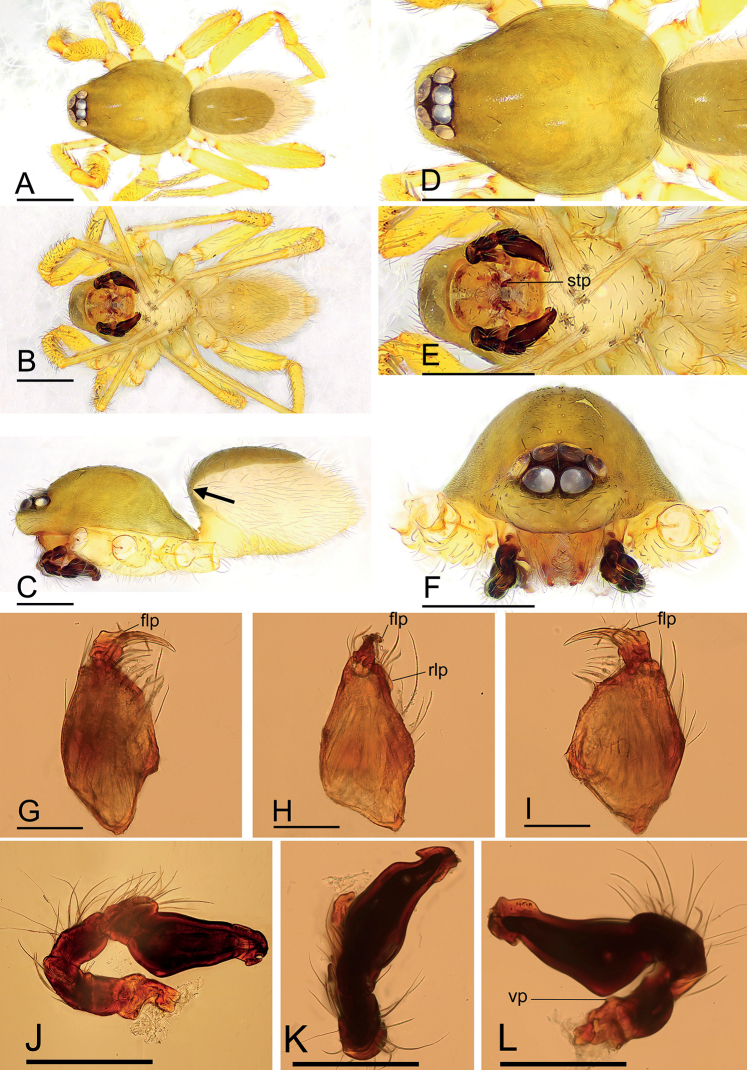
*Ischnothyreuspome* sp. nov., male holotype **A–C** habitus in dorsal, ventral and lateral views (arrow shows the fused scuta) **D–F** prosoma, dorsal, ventral and anterior views **G–I** left chelicera, anterior, lateral and posterior views **J–L** left palp, prolateral, dorsal and retrolateral views. Abbreviations: flp = flag-like process; rlp = ridge-like protuberance; stp = strong, tooth-like projection; vp = ventral projection. Scale bars: 0.4 mm (**A–F**); 0.1 mm (**G–L**).

**Figure 7.**
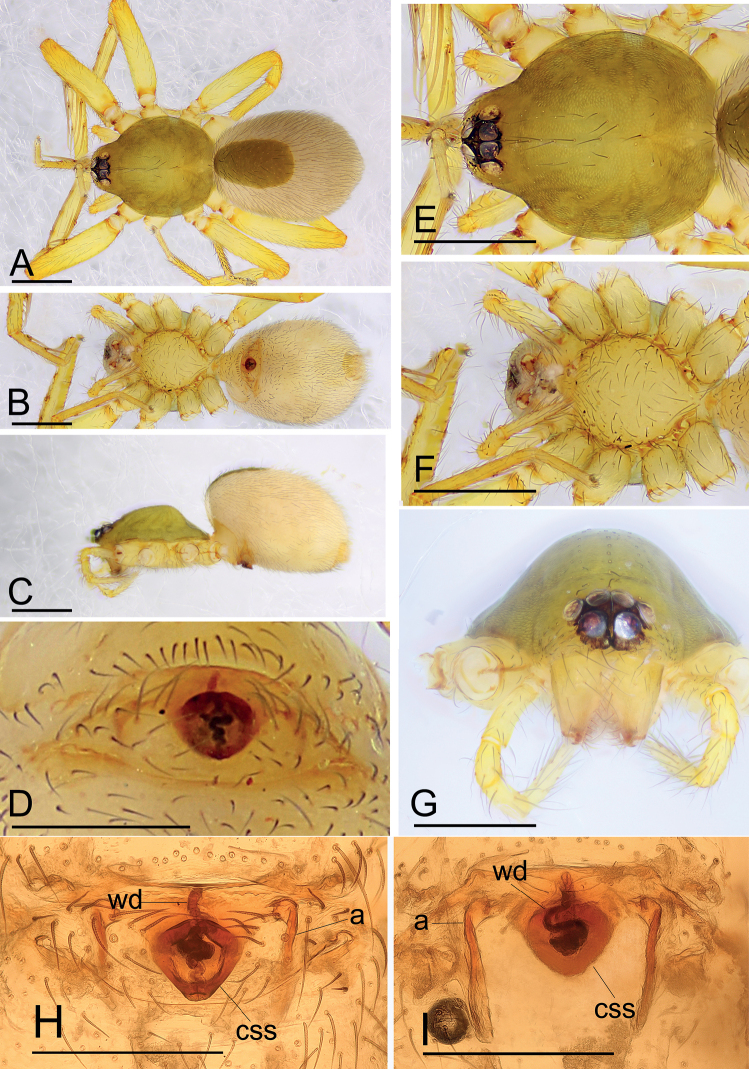
*Ischnothyreuspome* sp. nov., female paratype **A–C** habitus in dorsal, ventral and lateral views **D** epigastric region, ventral view **E–G** prosoma, dorsal, ventral and anterior views **H, I** endogyne, ventral and dorsal views (cleared in lactic acid). Abbreviations: a = apodeme; css = chestnut-shaped structure; wd = winding duct. Scale bars: 0.4 mm (**A–C, E–G**); 0.2 mm (**D, H, I**).

Figs 4D–F , 6A–L , 7A–I

##### Type material.

***Holotype*** ♂ (SYNU-526): China, Tibet, Nyingchi, Pome County, road to Metok County, 80 K; 29°39.897'N, 95°29.963'E; 2140±5 m; 10.VIII.2013; Qi Cao leg. ***Paratypes*** 2♂, 3♀ (SYNU-527-531): same data as for holotype.

##### Diagnosis.

Females of the new species are similar to those of *I.jianglangi* Tong & Li, 2020 in having the large, chestnut-shaped structure of the endogyne, but can be distinguished by the simple winding duct of the endogyne (Fig. 7H, I) vs. the complex winding duct (see Tong et al. 2020: fig. 17B). Males of the new species are similar to those of *I.yunlong* Tong & Li, 2021 in having the large flag-like sclerotized process of the cheliceral fang, but can be distinguished by the fused abdominal dorsal and epigastric scuta (Fig. 6C) vs. unfused (see Huang et al. 2021: fig. 1E), and by lacking the dorsal protuberance on the distal end of the bulb (Fig. 4D, E) vs. with dorsal protuberance (see Huang et al. 2021: fig. 2J).

##### Description.

**Male (holotype). *Body***: habitus as in Fig. 6A–C; body length 1.58. ***Carapace***: 0.84 long, 0.69 wide; yellow, oval in dorsal view, with brown egg-shaped patches behind eyes, pars cephalica strongly elevated in lateral view, surface of elevated portion of pars cephalica smooth, sides finely reticulate, lateral margin straight, smooth (Fig. 6D). ***Clypeus***: straight in frontal view, ALE separated from edge of carapace by 1.4× of their diameter (Fig. 6F). ***Eyes***: ALE largest, ALE circular, PME squared, PLE oval; posterior eye row recurved from above; ALE touching, ALE-PLE touching (Fig. 6D, F). ***Sternum***: as long as wide, pale orange (Fig. 6E). ***Mouthparts***: chelicerae, endites, and labium yellow; chelicerae straight, with ridge-like protuberance at anterior face, base of fangs with large flag-like sclerotized process, fang groove with a few small and two larger denticles (Figs 4F, 6G–I); anteromedian tip of endites with one strong, tooth-like projection (Fig. 6E). ***Abdomen***: 0.74 long, 0.46 wide; dorsal scutum well sclerotized, dark brown, covering 3/5 of abdomen width and approximately 2/3 of abdomen length, fused to epigastric scutum (arrow in Fig. 6C); postgastric scutum covering ~ 5/6 of abdomen length. ***Legs***: pale orange, femur I with two prolateral spines, tibia I with four pairs, metatarsus I with two pairs of long ventral spines. Leg II spination similar to leg I, except femur with only one prolateral spine. Legs III and IV spineless. ***Palp***: trochanter with ventral projection; bulb without small ventral protuberance, distal end of bulb stout, with needle-like membrane and broad leaf-like projection (Figs 4D, E, 6J–L).

**Female (paratype, SYNU-529).** Same as male except as noted. ***Body***: habitus as in Fig. 7A–C; body length 1.64. ***Carapace***: 0.77 long, 0.67 wide. ***Mouthparts***: chelicerae and endites unmodified. ***Abdomen***: 0.88 long, 0.65 wide; dorsal scutum very small; epigastric scutum well sclerotized, orange; postgastric scutum widely hexagonal, only around epigastric furrow. ***Endogyne***: winding duct simple, with anterior portion straight, strongly convoluted only in posterior section, ending in large, chestnut-shaped structure (Fig. 7H, I).

##### Etymology.

The specific name is a noun in apposition taken from the type locality.

##### Distribution.

Known only from the type locality.

## Supplementary Material

XML Treatment for Ischnothyreus caoqii

XML Treatment for Ischnothyreus linzhiensis

XML Treatment for Ischnothyreus metok

XML Treatment for Ischnothyreus pome
